# Genome-Wide Analyses of Proteome and Acetylome in *Zymomonas mobilis* Under N_2_-Fixing Condition

**DOI:** 10.3389/fmicb.2021.740555

**Published:** 2021-10-07

**Authors:** Ayesha Nisar, Xiangxu Gongye, Yuhuan Huang, Sawar Khan, Mao Chen, Bo Wu, Mingxiong He

**Affiliations:** ^1^Key Laboratory of Development and Application of Rural Renewable Energy (Ministry of Agriculture and Rural Affairs), Biogas Institute of Ministry of Agriculture and Rural Affairs, Chengdu, China; ^2^Graduate School of Chinese Academy of Agricultural Science, Beijing, China

**Keywords:** *Zymomonas mobilis*, 4D label-free proteomics, nitrogen fixation, post-translational modification, lysine acetylome

## Abstract

*Zymomonas mobilis*, a promising candidate for industrial biofuel production, is capable of nitrogen fixation naturally without hindering ethanol production. However, little is known about the regulation of nitrogen fixation in *Z. mobilis*. We herein conducted a high throughput analysis of proteome and protein acetylation in *Z. mobilis* under N_2_-fixing conditions and established its first acetylome. The upregulated proteins mainly belong to processes of nitrogen fixation, motility, chemotaxis, flagellar assembly, energy production, transportation, and oxidation–reduction. Whereas, downregulated proteins are mainly related to energy-consuming and biosynthetic processes. Our acetylome analyses revealed 197 uniquely acetylated proteins, belonging to major pathways such as nitrogen fixation, central carbon metabolism, ammonia assimilation pathway, protein biosynthesis, and amino acid metabolism. Further, we observed acetylation in glycolytic enzymes of central carbon metabolism, the nitrogenase complex, the master regulator NifA, and the enzyme in GS/GOGAT cycle. These findings suggest that protein acetylation may play an important role in regulating various aspects of N_2_-metabolism in *Z. mobilis*. This study provides new knowledge of specific proteins and their associated cellular processes and pathways that may be regulated by protein acetylation in *Z. mobilis*.

## Introduction

*Zymomonas mobilis*, a Gram-negative facultative anaerobic bacterium, is a promising candidate for industrial biofuel production with a suite of desirable characteristics (Doelle et al., 1993; Sprenger, 1996; Kalnenieks, 2006; Panesar et al., 2006; Bai et al., 2008; Yang et al., 2009, 2016; He et al., 2014; Martien et al., 2019; Todhanakasem et al., 2020). *Zymomonas mobilis* is capable to fix dinitrogen (N_2_) efficiently without affecting ethanol production, which further increases its economic and environmental importance (Kremer et al., 2015; Palamae et al., 2020). In comparison with other free-living diazotrophic bacteria, *Z. mobilis* has just a Mo-dependent nitrogen-fixing system. The nitrogen-fixing island of the ZM4 strain, for example, is a 23-kb chromosomal region that contains the transcriptional regulator *nifA*, the *rnf* operon encoding the Rnf electron transport complex, the nitrogenase operon, and two operons involved in nitrogenase maturation (Tsoy et al., 2016; Tatli et al., 2019).

Aside from the conserved genetic structure of the nitrogen-fixing island, it is also very important to decipher the regulatory mechanisms underlying the nitrogen fixation. This will not only help to better understand nitrogen fixation but is also crucial for developing more efficient nitrogen-fixing pathways. Such novel pathways will help in reducing the use of chemical fertilizers and replacing the nitrogen source in the fermentation industry. Nitrogen fixation in free-living diazotrophic bacteria is regulated at diverse levels, including transcriptional regulation by NifA (or VnfA) and its regulator NifL (Dixon, 1998; Lei et al., 1999; Schmitz et al., 2002; Martinez-Argudo et al., 2004b; Perry et al., 2005), post-transcriptional regulation by non-coding RNAs (Wang et al., 2009; Zhan et al., 2016, 2019), translational regulation by a conformational switch in NifL (Martinez-Argudo et al., 2004a), and various post-translational modifications (PTMs). In addition, nitrogen fixation is also regulated by sensing oxygen, ammonium, cofactors, or signal messengers (Dixon and Kahn, 2004; Masepohl and Hallenbeck, 2010; Xie et al., 2010; Zhang et al., 2012; Hoffmann et al., 2015).

The PTM-based regulation of nitrogen fixation is of great importance. For instance, phosphorylation can activate the Ntr two-component system, which enhances nitrogen fixation (Jiang et al., 1998; Marx et al., 2016). ADP-ribosylation, which is mediated by DraT (ADP-ribosyltransferase) and DraG (ADP-ribosyl glycohydrolase), is another prevalent regulatory mechanism in diazotrophs (Huergo et al., 2012; Nordlund and Högbom, 2013; Moure et al., 2015; Bueno Batista and Dixon, 2019). Furthermore, uridylylated PII protein (GlnK)-mediated physiological switch-off of nitrogenase activity *via* inhibition of the Rnf1 electron transport system is also a known regulatory mechanism (Sarkar et al., 2012; Bueno Batista and Dixon, 2019). The *Rhodospirillum rubrum* mutants of GlnD (a bifunctional uridylyltransferase/uridylyl-removing enzyme) affected nitrogen fixation in various ways (Zhang et al., 2005). These studies emphasize the significance of uridylylation in regulation of nitrogen fixation.

Protein acetylation is one among many PTMs used to regulate cellular processes across all domains of life. Acetylation can dramatically change the protein’s function by altering its properties such as solubility, hydrophobicity, surface properties, and stability. This, in turn, may influence protein conformation and interactions with other macromolecules such as DNA, other proteins, substrates, and cofactors, among others (Glozak et al., 2005; Yang and Seto, 2008; Xiong and Guan, 2012). While protein acetylation is well known for its regulatory role in eukaryotes (Hebbes et al., 1988; Glozak et al., 2005; Kim et al., 2006; Choudhary et al., 2009; Zhao et al., 2010; Drazic et al., 2016; Ree et al., 2018), research about the role of this PTM in prokaryotes is relatively a recent scientific endeavor (Yu et al., 2008; Hentchel and Escalante-Semerena, 2015; Ouidir et al., 2016; Wolfe, 2016; Carabetta and Cristea, 2017; Nakayasu et al., 2017; Ren et al., 2017; Liimatta et al., 2018; Liu et al., 2018a; Reverdy et al., 2018; Christensen et al., 2019a,b; VanDrisse and Escalante-Semerena, 2019). So far, we know little about the role of acetylation in regulation of nitrogen fixation. A series of investigations, however, revealed that GlnR mediates an autofeedback-loop to regulate glutamine synthase activity *via* lysine-acetylation (You et al., 2016, 2017; Liu et al., 2018b). Based on these findings, acetylation is speculated to regulate the nitrogen fixation.

However, the regulation of N-fixation in *Z. mobilis* is not fully understood. Unlike many other diazothophs, ZM4 does not appear to encode NifL transcriptional regulator or DraT/DraG-mediated ADP-ribosylation system. In addition, its employs a NtrX-NtrY instead of a NtrB-NtrC two-component system to regulate the nitrogen fixation (Yang et al., 2018). These genomic traits indicate that there are specific regulations for nitrogen fixation in ZM4. At post-translational level, a recent phosphoproteomic study in *Z. mobilis* has revealed that various proteins involved in nitrogen fixation such as NifA, NtrC, PII proteins, Rnf, Hfq, and nitrogenase, were significantly phosphorylated (Tatli et al., 2019). However, ZM4’s proteomic profiling and other PTMs under nitrogen-fixing conditions are yet to be described. Therefore, in the present study, we conduct 4D label-free proteomics in *Z. mobilis* strain ZM4 under N_2_-fixing and non-fixing conditions. We describe the first genome-wide proteome and acetylome in this ethanologenic bacterium.

## Materials and Methods

### Strain and Growth Conditions

A single colony of *Z. mobilis* (sub sp. mobilis ZM4 strain) was inoculated to 20ml of the rich media (RM; 20g/l glucose, 10g/l yeast extract, 1g/l KH_2_PO_4_, 1g/l peptone, and 1g/l MgSO_4_•7H_2_O) and then cultured for 20h (OD600 ~1.5 to 1.9) in a 50-ml screw-cap tube. Then 10ml of the cultures were transferred in 100ml of RM and grown for 20h in a 250-mL Duran bottle. Cells were harvested by centrifuging at 1216 RCF and 4°C for 10min (Sorvall BIOFUGE Stratos, Thermo Scientific), washed twice with autoclaved ddH_2_O, and suspended in 100ml autoclaved ddH_2_O. Twelve milliliters of the suspended cells were inoculated in 120ml of nitrogen-fixing media, the modified ZYMM (Kremer et al., 2015), in a 300-ml serum bottle, followed by flushing nitrogen gas (N_2_, with the purity of 99.999%) at 1MPa for 30min. Then the serum bottle was sealed and incubated at 30°C with shaking at 150rpm. Twenty-four-hour cultures (N-fixing) were centrifuged at 1751 RCF and 4°C for extraction of cellular proteins. The modified ZYMM used in this study was similar to that of Kremer et al. (2015), with the exception that ammonium molybdate was replaced with sodium molybdate. For non-fixing condition, the ZM4 strain was grown in non-nitrogen-fixing media (10mM ammonium chloride instead of N_2_ was used in the modified ZYMM), and samples were taken at exponential phase (8h culture).

### Protein Extraction and Digestion

The samples were sonicated three times on ice in lysis buffer (8M urea, 1% Protease Inhibitor Cocktail, 3μM trichostatin A, and 50mM nicotinamide) using a high-intensity ultrasonic processor (Scientz). Debris was removed by centrifugation (12,000g at 4°C for 10min). Supernatants were collected and precipitated with cold 20% trichloroacetic acid for 2h on ice. Samples were centrifuged (12,000g at 4°C for 5min), supernatants were discarded, and the pellets were washed three times with cold acetone. The protein samples were redissolved in 8M urea, and protein concentration was determined with a BCA Kit (Beyotime) as per the manufacturer’s instructions.

For trypsin digestion, the protein samples (after washed with cold acetone) were redissolved in buffer (100mM TEAB, pH 8.0). Then trypsin was added at a 1:50 ratio and samples were incubated overnight. Trypsin was added to the samples again at a 1:100 ratio, and the samples were incubated for 4h. Finally, the samples were reduced with 5mM DTT (for 45min at 37°C) and alkylated with 15mM iodoacetamide (for 45min at RT in the dark).

### Enrichment of Acetylated Lysine Containing Peptides

To enrich the acetylated peptides, the tryptic peptides were dissolved in NETN buffer (100mM NaCl, 1mM EDTA, 50mM Tris–HCl, 0.5% NP-40, pH 8.0), and samples were incubated (overnight at 4°C) with pre-washed antibody beads (PTM-104, PTM Bio) with gentle shaking. The beads were then washed four times with NETN buffer and twice with water. The bound peptides were eluted from the beads with 0.1% trifluoroacetic acid. Finally, the eluted fractions were combined and vacuum-dried. For LC–MS/MS analysis, the resulting peptides were desalted with C_18_ ZipTips (Millipore) according to the manufacturer’s instructions.

### LC–MS/MS Analysis

The tryptic peptides were dissolved in buffer A (0.1% formic acid in water) and were directly loaded onto a reversed-phase analytical column (25-cm length, 75μm i.d.). Peptides were eluted at a constant flow rate of 400nl/min on a nanoElute UHPLC system (Bruker Daltonics), using a 3-step linear gradient: from 6 to 22% buffer B (0.1% formic acid in acetonitrile) over 43min, from 22 to 30% B in 13min, climbing to 80% B in 2min, and then holding at 80% B for the last 2min. The mass spectrometry was performed with timsTOF Pro (Bruker Daltonics) instrument with an electrospray voltage of 1.60kV. Precursors and fragments were analyzed at the TOF detector with an MS/MS scan range from 100 to 1700m/z. The timsTOF Pro was operated in parallel accumulation serial fragmentation (PASEF) mode. Precursors with charge states 0–5 were selected for fragmentation, and 10 PASEF-MS/MS scans were acquired per cycle. The dynamic exclusion was set to 30s.

### Database Search and Data Analysis

The resulting MS/MS data were processed using MaxQuant software v.1.6.6.0.^1^ Tandem mass spectra were searched against the database (Zymomonas_mobilis_subsp._Mobilis_strainATCC_31821_264203_PR_ 20190830) and concatenated with reverse decoy database. Trypsin/P was specified as a cleavage enzyme allowing up to four missing cleavages. The mass tolerance for precursor ions was set as 40ppm in the First search and 40ppm in the Main search, while the mass tolerance for fragment ions was set as 0.04Da. Carbamidomethyl on Cys was specified as fixed modification, while acetylation on protein N-terminal, oxidation on Met, and acetylation on Lys were specified as variable modifications. FDR was adjusted to <1%. The intensity values for each acetylated site were averaged across the biological replicates for each growth condition. A particular comparison ratio of average scores between the two conditions was considered as a fold change of comparison at a specific acetylated site. Acetylated sites that were not quantified in at least two biological replicates of each condition in a relevant comparison were excluded from analyses. Data were subjected to log2 transformation. A two-tailed student’s T-test was performed for each comparison at a specific acetylated site. A value of *p*<0.05 was considered significant. All value of *p*s were then converted to -log10 scores.

### Bioinformatics Annotation of Peptides

All identified peptides were subjected to Gene Ontology (GO) annotations and enrichment at the UniProt-GOA database.^2^ Proteins were classified by GO annotations under three categories (biological process, cellular component, and molecular function). For each category, a two-tailed Fisher’s exact test was employed, and a GO term with a value of *p*<0.05 was considered as significant. For annotation of metabolic pathways, the Kyoto Encyclopedia of Genes and Genomes (KEGG; Kanehisa and Goto, 2000; Tanabe and Kanehisa, 2012) was used. Protein interactions networks were generated using the STRING database with an interaction confidence score of 0.7 (Szklarczyk et al., 2015). Subcellular localization of proteins was predicted using CELLO v.2.5 (Yu et al., 2006). Significant values of *p* for each analysis were filtered and transformed into −log10 scores for use in graphics. The 3D structures of proteins were predicted using SWISS-MODEL server.^3^

## Results

### 4D Label-Free Quantitative Proteomics of *Zymomonas mobilis* Under N_2_-Fixing and Non-fixing Conditions

**Figure 1:**
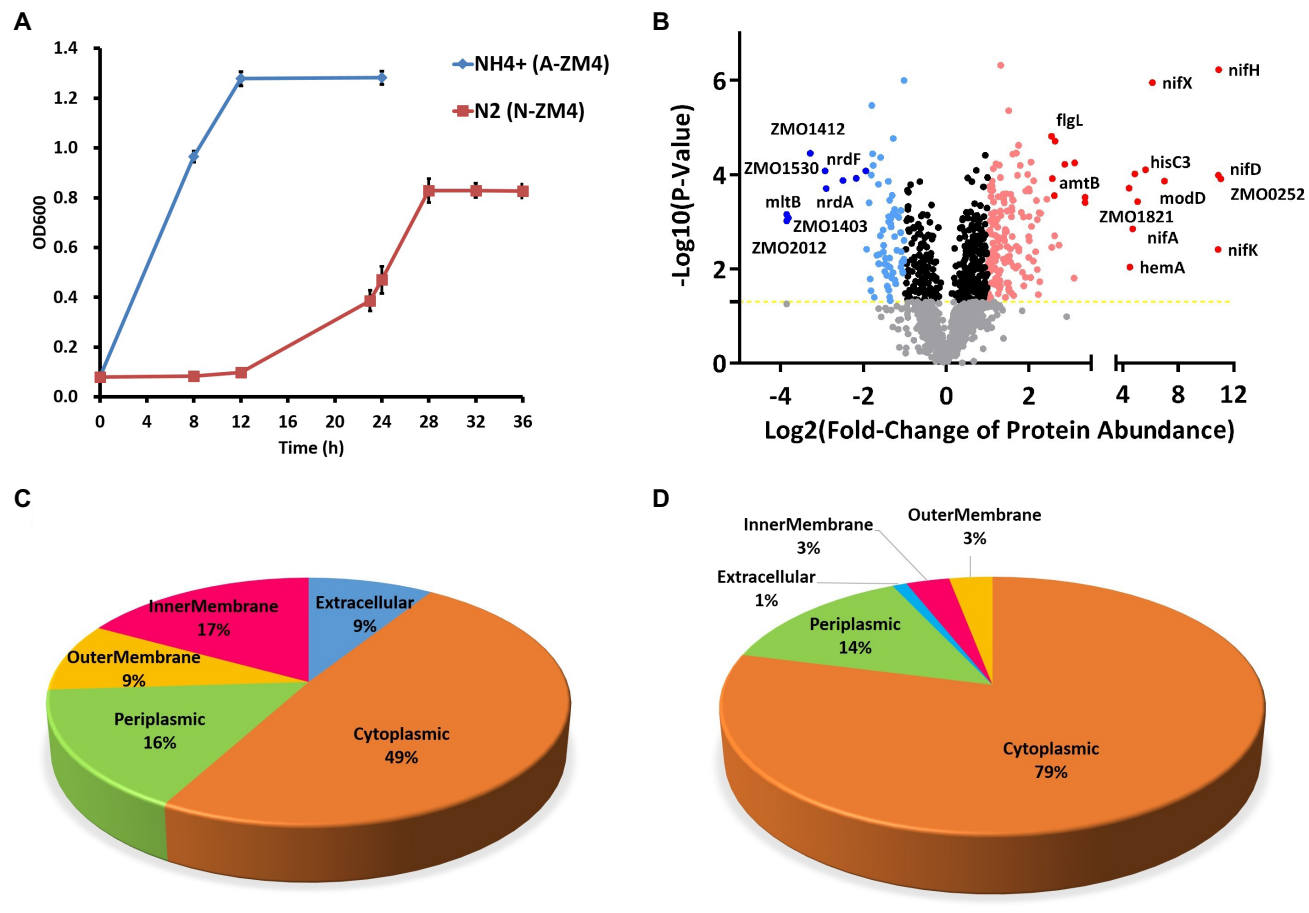
Proteomics analyses of *Zymomonas mobilis* under N_2_-fixing conditions. **(A)** Growth of *Z. mobilis* under the nitrogen-fixing (red) and non-fixing (blue) conditions. **(B)** Differential protein expression under N_2_-fixing conditions. Protein names are shown for some highly differentially expressed proteins. **(C)** Subcellular localization of upregulated proteins. **(D)** Subcellular localization of downregulated proteins.

Cells grown under the N_2_-fixing (N-ZM4) and non-fixing (A-ZM4) conditions (Figure 1A) were harvested during exponential growth and lysed for high-resolution LC–MS/MS analysis. The resulting identified peptides were quantitatively compared by label-free quantitation. We identified a total of 1,449 proteins, of which 1,389 proteins were quantifiable (Supplementary Table S1). Of the quantifiable proteins, 242 showed significant differences between nitrogen-fixing (N-ZM4) and non-fixing (A-ZM4) group (Figure 1B). Among these, 170 proteins were upregulated (fold change>2 with *p*<0.05), and 72 were downregulated (fold change<0.5 with *p*<0.05) under the nitrogen-fixing condition. All of these proteins were chromosome encoded except four proteins (three upregulated, and one downregulated) that were encoded by native plasmid pZM39 (GenBank ID NZ_CP023719.1). One of the pZM39-encoded upregulated proteins (Q8GF46) was an autotransporter domain-containing protein, and two (Q8GF51 and D3G2C3) were uncharacterized proteins. Whereas, the single pZmo1-encoded downregulated protein (Q8GF26) was a putative partitioning protein ParB.

Various proteins associated with N2-fixation were highly upregulated under N2-fixing condition, including nitrogenase complex (NifH, NifD, NifK, and NifX), the regulators of N2-fixation (NifA, GlnB, and GlnA), Rnf electron transport complex (RnfH, RnfC, and RnfD), nitrogen regulation sensor (NtrY), sigma factor (RpoN), ammonium transporter (AmtB), and molybdenum transport system protein (ModD).

Various proteins related to energy production and transportation of energy molecules were also upregulated (Supplementary Table S1). These include leavansucreases SacB and SacC, sugar transporters (Glf, OprB1, and ZMO0293), ATPase (ZMO0637), and a predicted major intrinsic protein (ZMO0252) that facilitates the passive transport of water, small carbohydrates, and other molecules. Similarly, TonB-dependent receptors and their associated proteins (ExbD and ExbB), which mediate substrate-specific transport across the outer membrane were also upregulated. The ABC transporter proteins ZMO0910, ZMO0254, ZMO1017, and ZMO1018 were also upregulated.

Proteins involved in motility, chemotaxis, and flagellar assembly were also found upregulated. Among other upregulated proteins the alcohol dehydrogenase was also included, implying that N_2_-fixation does not affect ethanol production. Proteins related to ferrous Fe^2+^ ion transport and its insertion into protoporphyrin IX, iron–sulfur cluster assembly, magnesium transporter, and potassium transporter were also upregulated. Proteins involved in the oxidation–reduction process and suppression of deoxyribonucleotides synthesis, for example NrdR, were upregulated too.

Downregulated proteins were mainly associated with energy-consuming processes and biosynthesis of secondary metabolites. Examples include blockage of malonyl-CoA-based biosynthesis, sesquiterpenoid and triterpenoid biosynthesis, biotin biosynthesis, folate biosynthesis, and ammonia synthesis from glycine. Conversion of Fe^2+^ to Fe^3+^ ions was also inhibited (downregulated bacterioferritin) during N_2_-fixation. Whereas, Fe^2+^ ion utilizing proteins were upregulated.

To further analyze which cellular areas were affected by differential protein expression, we performed subcellular localization analysis of differentially expressed proteins. Among the upregulated proteins, 49% were localized to cytoplasmic, 16% to periplasmic, 17% to the inner membrane, 9% to the outer membrane, and 9% to extracellular categories (Figure 1C). Whereas, localization of downregulated proteins resulted in 79% to cytoplasmic, 14% to periplasmic, 3% to the inner membrane, 3% to the outer membrane, and 1% to extracellular categories (Figure 1D).

### Functional Enrichment of Differentially Expressed Proteins

We then further focused on differentially expressed proteins and performed functional enrichment of associated GO terms and pathways, and visualized significantly enriched GO terms and KEGG pathways under both conditions.

**Figure 2:**
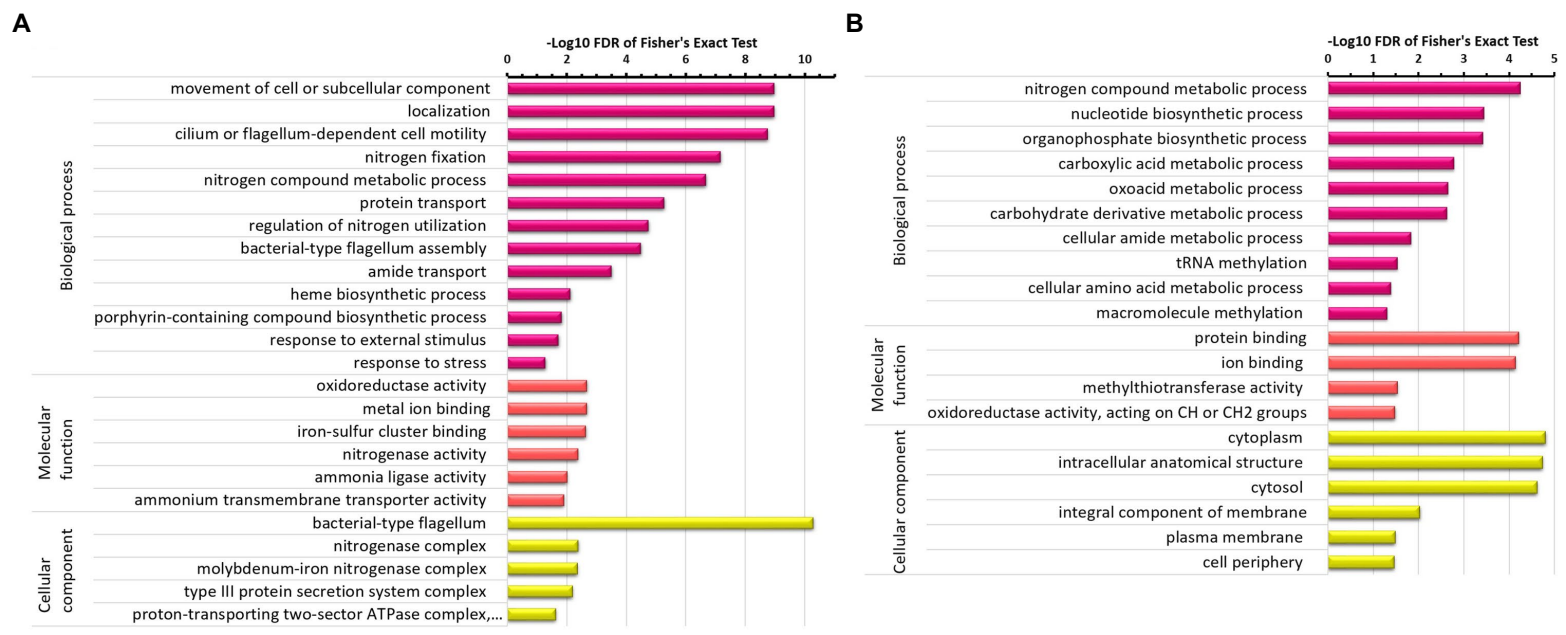
GO terms enrichment analysis of differentially expressed proteins in *Z. mobilis* under N_2_-fixing condition. **(A)** GO terms enrichment analysis of upregulated proteins under the N_2_-fixing condition. **(B)** GO terms enrichment analysis of downregulated proteins under the N_2_-fixing condition.

The significantly enriched areas for upregulated proteins (Figure 2A) under the N_2_-fixing condition were associated with motility of cell (flagellum-dependent) or subcellular component and their localization, nitrogen fixation, molecular transportation, and response to stress or external stimuli. Nitrogen fixation is a stressful condition and significantly enriched GO terms associated with response to stress or external stimuli can be seen here. The chemotaxis and cell motility are thus apparent. Various GO terms associated with nitrogen fixation process such as nitrogenase complex and its associated proteins like molybdenum-iron transporters, proton-transporting two-sector ATPase complex, heme, and porphyrin-containing compound biosynthesis, nitrogen compound metabolic process, regulation of nitrogen utilization, and transport of nitrogen compound were significantly enriched. Whereas, nitrogen compound metabolic process, nucleotide biosynthetic process, carboxylic acid metabolism, oxoacid metabolism, cellular amide metabolism, amino acid metabolism, and macromolecule methylation were significantly enriched areas for downregulated proteins (Figure 2B).

**Figure 3:**
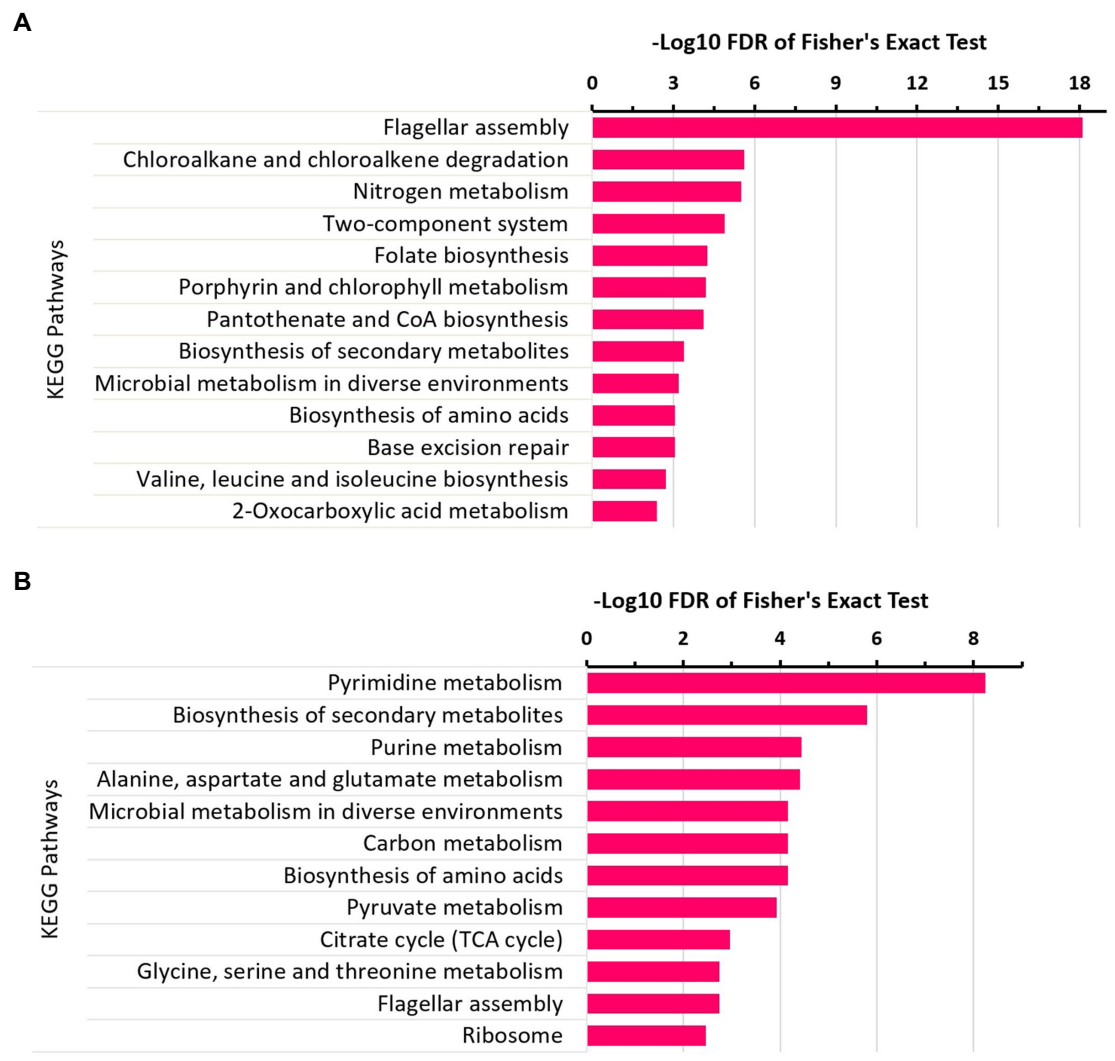
KEGG pathways enrichment analysis of differentially expressed proteins in *Z. mobilis* under N_2_-fixing condition. **(A)** KEGG pathways enrichment analysis of upregulated proteins under the N_2_-fixing condition. **(B)** KEGG pathways enrichment analysis of downregulated proteins under the N_2_-fixing condition.

Major KEGG pathways that were significantly enriched for upregulated proteins (Figure 3A) under the N2-fixing condition included the flagellar assembly, nitrogen metabolism, chloroalkane, and chloroalkene degradation, two-component system, folate biosynthesis, pantothenate and CoA biosynthesis, porphyrin and chlorophyll metabolism, and microbial metabolism in diverse environments. Some significantly enriched KEGG pathways for downregulated proteins (Figure 3B) were pyrimidine and purine metabolism, biosynthesis of secondary metabolites, carbon metabolism, and biosynthesis of amino acids.

### Acetylome Analysis of *Zymomonas mobilis*

The result of the above proteomics analysis revealed that ZMO1821, a protein located in ZM4’s nitrogen-fixing island, was upregulated along with other nitrogen-fixing associated proteins (Figure 1B). This protein was predicted as a SIR2 family protein that likely functions as a deacetylase. Since acetylation widely occurs in bacteria, we next conducted acetylome analyses of ZM4 under the nitrogen-fixing condition.

For acetylome analyses, the tryptic peptides obtained from each of the replicates were enriched for acetylated lysine residues and were then subjected to a high-resolution LC–MS/MS analysis on a timsTOF Pro instrument. The resulting identified peptides were quantitatively compared by label-free quantitation. We identified a total of 2,935 acetylation sites that were confidently localized (≥75% probability) across the samples (Supplementary Table S2). Of these, 1,591 sites had quantification information in at least two replicates of the comparison, and these sites were sorted into 663 unique acetylated proteins (Supplementary Table S3). In each protein, the number of acetylated sites varied from 1 to 16. Acetylome of *Z. mobilis* encompassed 37% of all of its proteins.

### Differential Protein Acetylation

**Figure 4:**
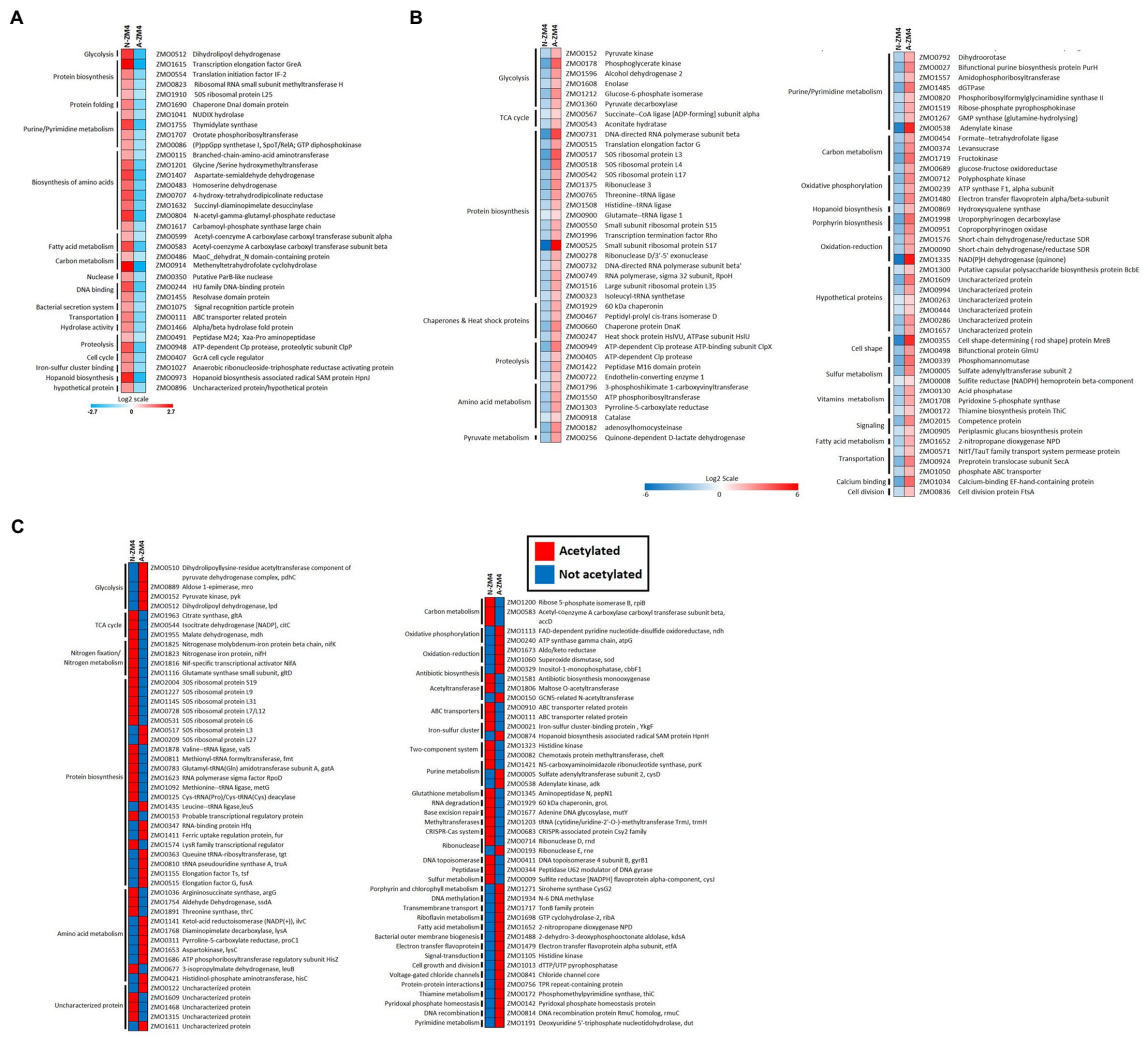
Differential protein acetylation in *Z. mobilis* under N_2_-fixing and non-fixing conditions. **(A)** Heatmap showing the comparative acetylation quantitation for 34 uniquely acetylated proteins under N_2_-fixing condition. **(B)** Heatmap showing the comparative acetylation quantitation for 83 uniquely acetylated proteins under non-fixing condition. For **(A,B)**, comparison were made between A-ZM4 and N-ZM4 and changes in proteins acetylation (fold-change, FC) are shown on the log2 fold scale, and quantitative data represent the average of three biological replicates. All quantitations were significant (*p*<0.05). **(C)** Heatmap showing non-FC-based protein acetylation across the growth conditions. For proteins that did not show relative changes in protein acetylation, the FC-based comparisons were not made; instead, changes in protein acetylation status are shown as follows: Red color indicates that protein is acetylated, Blue color indicates that protein is not acetylated.

To get insights into differential protein acetylation across the N_2_-fixing and non-fixing conditions, we compared the protein acetylation data among the different growth conditions. Our results showed that 197 proteins were uniquely acetylated at 229 sites across the growth conditions (Supplementary Table S4). Among these, 117 proteins (at 137 sites) showed relative changes in protein acetylation, and we made fold-change (FC) comparisons for these proteins (Figures 4A,B). In FC-based comparison, 34 proteins were uniquely acetylated (at 39 sites) under the N_2_-fixing condition (Figure 4A), while 83 proteins were uniquely acetylated (at 98 sites) under the non-fixing condition (Figure 4B). For proteins that did not show relative changes in protein-acetylation (i.e., significant acetylation was observed under the one growth condition only), the FC-based comparisons were not made, and protein-acetylation was simply considered as present or absent for such proteins. A total of 92 sites showed non-FC-based pattern of protein-acetylation across the growth conditions (Figure 4C). The master regulator of the nitrogen-fixing island (NifA), the nitrogenase subunits (NifH and NifK), and ammonia assimilatory protein glutamate synthase were found acetylated under the nitrogen-fixing condition (Figure 4C).

### Functional Enrichment of Differentially Acetylated Proteins

**Figure 5:**
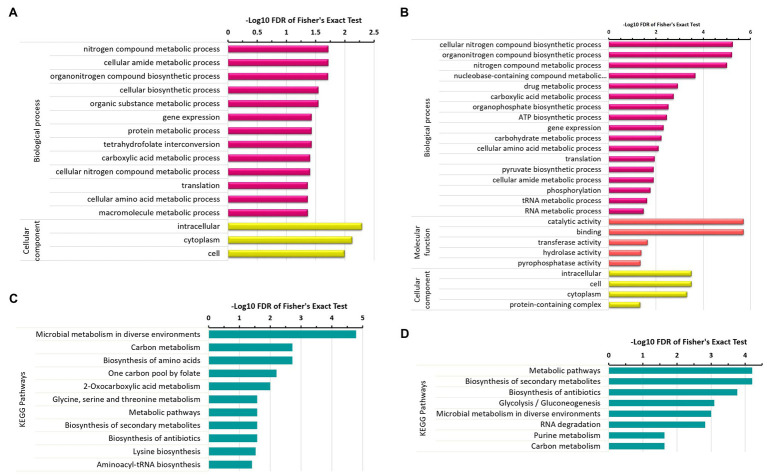
Enrichment analysis of differentially acetylated proteins in *Z. mobilis* under N_2_-fixing and non-fixing conditions. **(A)** GO terms enrichment analysis of uniquely acetylated proteins of N_2_-fixing condition. **(B)** GO terms enrichment analysis of uniquely acetylated proteins of non-fixing condition. **(C)** KEGG pathways enrichment analysis of uniquely acetylated proteins of N_2_-fixing condition. **(D)** KEGG pathways enrichment analysis of uniquely acetylated proteins of non-fixing condition.

To further explore which GO terms and pathways are significantly represented by these differentially acetylated proteins, we executed enrichment analysis of GO terms and KEGG pathways associated with differentially acetylated proteins. This allowed us to further narrow down our focus around the areas that were significantly affected by protein acetylation across the experimental conditions. Under the N_2_-fixing condition, the significantly enriched GO terms were nitrogen compound metabolic process, gene expression, protein metabolism, amide metabolism, carboxylic acid metabolism, and amino acid metabolism (Figure 5A). While the significantly enriched GO terms under the non-fixing condition were nitrogen compound metabolism, nucleobase-containing compounds metabolism, translation, ATP metabolic process, amide biosynthetic process, RNA metabolic process, organophosphate biosynthetic process, translation, and phosphorylation (Figure 5B). Whereas, the pathways such as microbial metabolism in diverse environments, carbon metabolism, amino acid metabolism, one carbon pool by folate, and biosynthesis of secondary metabolites were significantly enriched under the N_2_-fixing condition (Figure 5C). While the significantly enriched pathways under the non-fixing condition were biosynthesis of antibiotics, glycolysis/gluconeogenesis, RNA degradation, and purine metabolism (Figure 5D).

**Figure 6:**
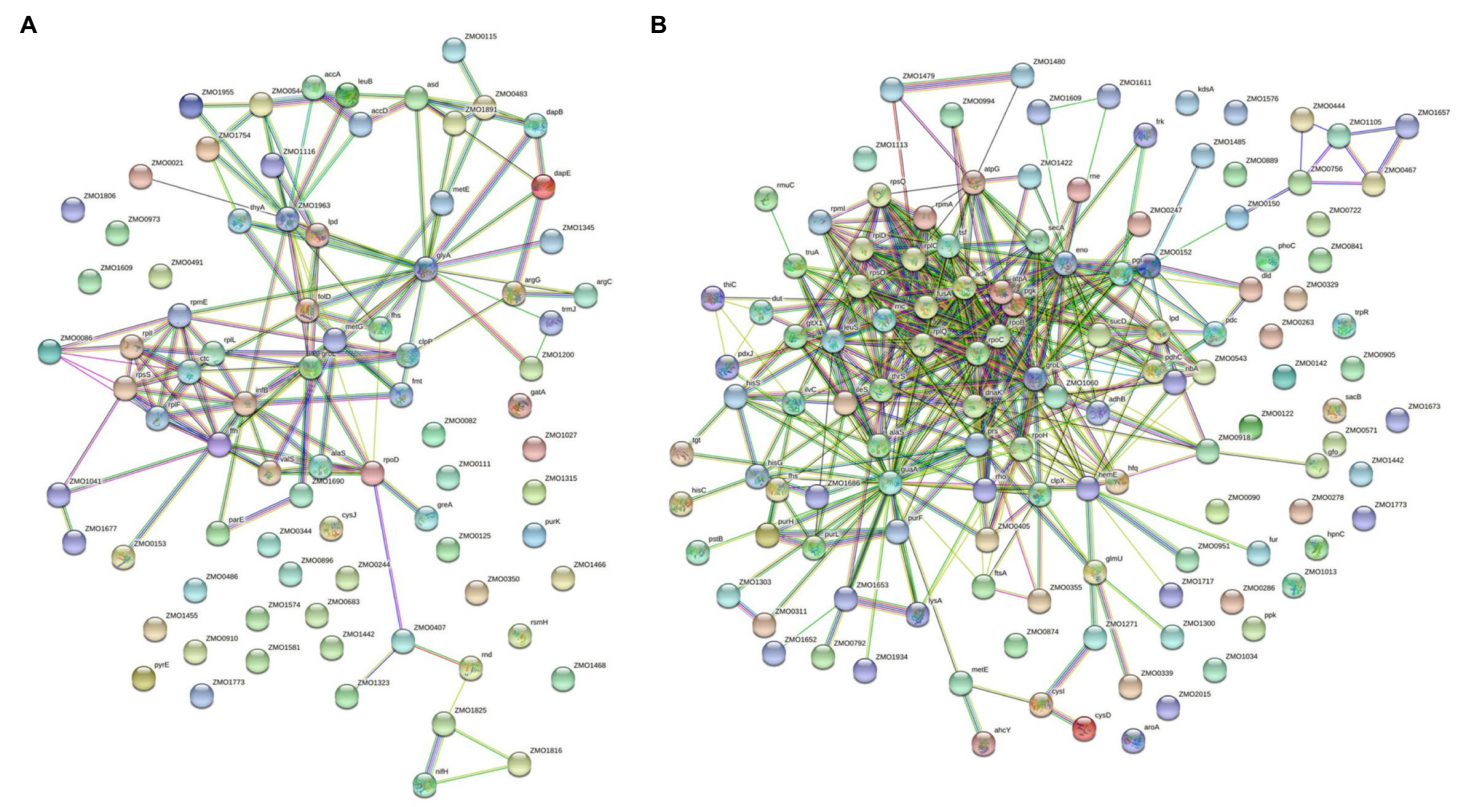
Protein interaction networks of differentially acetylated proteins in *Z. mobilis* under **(A)** N_2_-fixing condition and **(B)** non-fixing conditions.

To further explore the connections among all differentially acetylated proteins, we reconstructed protein interaction networks. Under the N_2_-fixing condition, there were 82 nodes with 136 predicted physical interactions (Figure 6A). Whereas, differentially acetylated proteins under the non-fixing condition formed 126 nodes that were connected by 434 predicted physical interactions (Figure 6B).

### Changes in Regulation and Acetylation of Proteins Associated With Central Carbon Metabolism and Nitrogen Fixation Pathway

**Figure 7:**
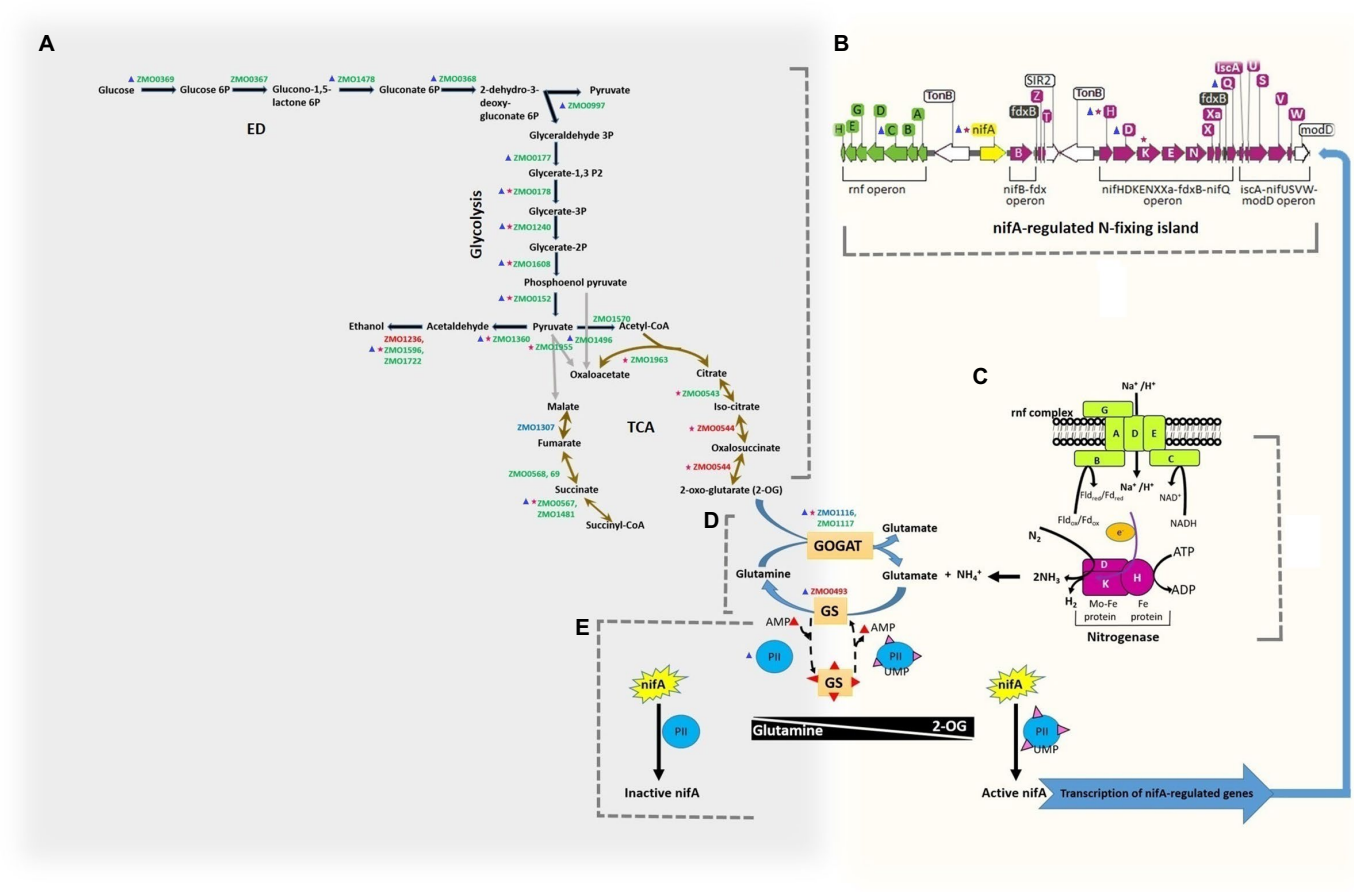
Regulation and acetylation of proteins in metabolic pathways of central carbon metabolism and nitrogen fixation in *Z. mobilis* in the present study. On the left side **(A)** the Entner–Doudoroff (ED) pathway, glycolysis, ethanol fermentation pathway and TCA cycle are shown. On the right side, *nifA*-regulated N-fixing island **(B)** and nitrogen fixation **(C)** are shown. Both of the metabolic circuits are connected in the center **(D)** by the ammonia assimilation pathway (GS/GOGAT cycle). At the bottom **(E)** the regulation of nitrogen fixation is depicted, where *nifA* acts as a master regulator to control the expression of nitrogen-fixing genes, and the activities of GS and *nifA* are putatively regulated by PII regulatory proteins. Whereas, concentrations of the regulatory metabolites glutamine and 2-oxoglutarate (2-OG) putatively regulate the interactions of PII proteins by their uridylylation (PII-UMP). The gene IDs in red color represent the up-regulated proteins, in blue color represents the downregulated proteins, and in green color represents the proteins that were not expressed differentially. The differentially acetylated proteins are shown with a small star in red color. The phosphorylated proteins (Tatli et al., 2019) are shown with a small triangle in blue color. All the pathways, reactions, and metabolites are consistent with the KEGG database record of *Z. mobilis*.

The pathway microbial metabolism in diverse environments includes nitrogen fixation and central carbon metabolism, and it was significantly enriched under nitrogen-fixing conditions. We, therefore, analyzed the nitrogen fixation in connection to central carbon metabolism for changes in regulation and acetylation of associated proteins (Figure 7). Further, to get a thorough understanding of nitrogen fixation in *Z. mobilis* at PTMs level, we also compared our results for protein phosphorylation under the N_2_-fixing condition with that of the previous study (Tatli et al., 2019) in *Z. mobilis*. The central carbon metabolism in *Z. mobilis* includes Entner–Doudoroff (ED) pathway, glycolysis, and incomplete TCA cycle (Figure 7A), which is linked to nitrogen fixation (Figures 7B,C; Bueno Batista and Dixon, 2019; Tatli et al., 2019) *via* ammonia assimilation pathway or GS/GOGAT cycle (Figure 7D; Bueno Batista and Dixon, 2019). Sugars are generally catabolized by *Z. mobilis via* the ED pathway into pyruvate and glyceraldehyde-3-phosphate (GA3P). The GA3P is ultimately converted into pyruvate by glycolysis. The *Z. mobilis* can convert >95% of the pyruvate into ethanol using pyruvate decarboxylase (Pdc) and alcohol dehydrogenases (AdhA and AdhB). We found that almost all of the proteins in ED pathway and glycolysis were not differentially expressed under the nitrogen-fixing condition. However, proteins such as phosphoglycerate kinase (Pgk), enolase (Eno), and pyruvate kinase (Pyk) were acetylated under the non-fixing condition. Pgk was acetylated at two positions (K244 and K271), Eno at one (K402), and Pyk at three (K51, K182, and K386). The protein malate dehydrogenase (Mdh) was acetylated under the N_2_-fixing condition at one site (K380). Whereas, a phosphoglycerate mutase (GpmA) was acetylated under both nitrogen-fixing (K126) and non-fixing (K104) conditions. In the ethanol-producing pathway, AdhA was upregulated and the rest of the proteins were normally regulated under the nitrogen-fixing condition. However, acetylation was also observed under the non-fixing condition in proteins Pdc (K219) and AdhB (K160) of the ethanol fermentation pathway. Recently, protein phosphorylation was observed (Tatli et al., 2019) in nearly all the glycolytic enzymes of central carbon metabolism in *Z. mobilis*. Among those, the KHG/KDPG aldolase (Eda), Eno, GpmA, and Pdc were phosphorylated under nitrogen-fixing condition, while AdhB was phosphorylated under the non-fixing condition.

In TCA cycle, isocitrate dehydrogenase (CitC, ZMO0544) was upregulated, fumarate hydratase (ZMO1307) was downregulated, and the rest of the proteins were normally regulated under the nitrogen-fixing condition. Whereas, two proteins of the TCA cycle were found acetylated under the non-fixing condition, that is, aconitate hydratase (AcnA, ZMO0543) at K663, and succinate–CoA ligase (SucD, ZMO0567) at K293. Also, CitC and GltA (ZMO1963) from TCA cycle were found acetylated at site K57 and K311 under the N_2_-fixing condition, respectively. In comparison, for phosphorylated-based PTM in *Z. mobilis*, only Mdh was reported phosphorylated among the TCA cycle proteins in the previous study (Tatli et al., 2019) under the nitrogen-fixing condition.

The ATP-dependent reduction of dinitrogen (N_2_) to bio-available ammonia (NH_3_) in *Z. mobilis* is carried out by molybdenum (Mo)-dependent nitrogenase complex encoded by the *nifH*, *nifD*, and *nifK* genes of N-fixing island (Figures 7B,C). We found that various proteins from the N_2_-fixing island such as NifA, NifH, NifD, NifK, NifX, ModD, RnfC, RnfD, and RnfH were highly upregulated under the nitrogen-fixing condition. However, only three N-fixing proteins, including NifA, NifH, and NifK, were significantly acetylated at K45, K12, and K395, respectively, under the N_2_-fixing condition. These acetylated lysine residues were found conserved by BLAST-p search in various diazotrophs species. To see if the acetylated residues of nitrogenase complex lie on the surfaces where the two nitrogenase proteins meet, we constructed hypothetical 3D structures of NifH and NifK of *Z. mobilis* and visualized the positions of acetylated K-residues, as shown in Supplementary Figures S1, S2. We also visualized the corresponding acetylated K-residues (for NifH and NifK) of *Z. mobilis* in the solved nitrogenase crystal structure of *Azotobacter vinelandii* (Supplementary Figure S3). The acetylated lysine of NifH (K12) was located inside its crystal structure near [4Fe-4S] clusters. However, it was not the point at which NifH met NifD or NifK. The acetylated lysine of NifK (K395) was located on the surface, and its corresponding K-residue in the nitrogenase structure was not found interacting with the other two proteins of the nitrogenase complex. Whereas, phosphorylation in *Z. mobilis* has been reported previously (Tatli et al., 2019) in proteins of N-fixing island under the nitrogen-fixing (NifH, NifD, NifQ, and RnfC) and non-fixing (NifA) conditions.

The nitrogen fixation and central carbon metabolism are connected by the ammonia assimilation pathway or GS/GOGAT cycle (Figure 7D), where fixed nitrogen is ultimately assimilated into central metabolism. The nitrogen regulatory protein PII (ZMO0492) putatively regulates ammonia assimilation and nitrogen fixation (Figure 7E). We observed that proteins GS and PII were upregulated. Whereas, ammonia assimilatory protein glutamate synthase (GltD, ZMO1116) was found acetylated under the N_2_-fixing condition at a single site (K27). Protein phosphorylation has also been reported under the nitrogen-fixing condition in GS, GOGAT, and PII proteins in the previous study (Tatli et al., 2019).

## Discussion

In this study, we conducted high throughput analyses of proteome and protein acetylation in *Z. mobilis* under N_2_-fixing and non-fixing conditions and established its first acetylome. We identified a total of 1,449 proteins, 242 of which were differentially and significantly regulated. Whereas, 229 sites of 197 proteins were uniquely acetylated across the growth conditions.

The genome-wide proteome analyses revealed that upregulated proteins were mostly involved in processes such as nitrogen fixation, motility, chemotaxis, flagellar assembly, energy production, transportation, and oxidation–reduction (Figures 2, 3). Since nitrogen fixation is a costly process that requires a supply of ATP and reducing power (Yan et al., 2010; Varley et al., 2015; Suyal et al., 2017), it is reasonable that the proteins involved in energy production or oxidation–reduction were upregulated under nitrogen fixation. Flagellar motility and chemotaxis have been demonstrated to be involved in energy taxis, a behavior that bacteria seek optimal conditions for metabolic activity, under the condition of nitrogen fixation (Xie et al., 2010; Ganusova et al., 2021). We hypothesize that the similar chemosensing and signaling machinery for nitrogen fixation exists in ZM4. Overall, our results show that *Z. mobilis* has the same molecular responses to nitrogen fixation as other diazotrophic organisms. A notable finding was that the ethanol-producing protein alcohol dehydrogenase was upregulated, which is consistent with prior studies showing that *Z. mobilis* can fix nitrogen efficiently without affecting ethanol production (Kremer et al., 2015; Palamae et al., 2020; Alencar et al., 2021).

We established the first-ever acetylome of *Z. mobilis* under nitrogen fixation. As shown in Figure 7, the acetylated proteins were significantly enriched in pathways such as nitrogen fixation, central carbon metabolism, and ammonia assimilation (GS/GOGAT cycle). Acetylation is a complex mechanism that can both upregulate and/or downregulate the activities of targeted proteins (Bontemps-Gallo et al., 2018). Acetylation in bacteria has been shown to modulate cellular metabolic processes or pathways ranging from transcription and translation to central metabolism (Hu et al., 2010; Wang et al., 2010; Bernal et al., 2014; Wolfe, 2016; Carabetta and Cristea, 2017; Nakayasu et al., 2017). Protein acetylation appears to be a prevalent and conserved mechanism in bacteria (Gardner and Escalante-Semerena, 2008; Wang et al., 2010; Crosby et al., 2012; Hayden et al., 2013; Tucker and Escalante-Semerena, 2013; Sang et al., 2016; Nakayasu et al., 2017; Bontemps-Gallo et al., 2018). Our results are consistent with these studies. Moreover, the nitrogen fixation and central carbon metabolism are linked by GS/GOGAT cycle (Bueno Batista and Dixon, 2019; Tatli et al., 2019), where fixed nitrogen is ultimately assimilated into central metabolism. Thus, the acetylome of *Z. mobilis* under the nitrogen-fixing condition provides a further extension that acetylation in bacteria can regulate both carbon and nitrogen metabolism. Furthermore, we also detected acetylation in the nitrogenase complex and the master regulator NifA under the nitrogen fixation. The acetylated lysine residues of these proteins were found to be conserved among the diazotrophs. Therefore, it is reasonable to speculate that protein acetylation plays a key role in regulating nitrogen metabolism and nitrogen fixation in *Z. mobilis*.

Therefore, an intriguing question arises: what enzymes can regulate the protein acetylation in *Z. mobilis*? By searching the ZM4 genome, three hypothetical SIR2 family proteins (Yang et al., 2018), a main lysine deacetylase family, were found. One of them is located on chromosome, and the other two are encoded by a native plasmid pZM32. Interestingly, the hypothetical chromosomal SIR2 encoding gene (*ZMO1821*) is located in the nitrogen-fixing island between *nifA* and nitrogenase genes (*nifH*, *nifD*, and *nifK*). Our proteomic analysis showed that the ZMO1821 protein, NifA, and nitrogenase genes were upregulated under the nitrogen-fixing condition (Figure 1B and Supplementary Table S1). This result motivated us to investigate a lysine acetylome profiling in ZM4. Moreover, we find that the acetylation status of several putative acetyltransferases, such as ZMO0150, ZMO1806, and ZMO1400, was changed under nitrogen fixation. These acetyltransferases are most likely involved in reversible acetylation reactions with SIR2 deacetylases. Combined with the phosphorylation proteome profiling, NifA, NifH, and NifK were both phosphorylated and acetylated under the nitrogen-fixing condition (Figure 7). Thus, we assume there is a regulation interaction between the both PTMs. In other prokaryotes, the SIR2 family protein CobB and its orthologs have been extensively studied. They perform diverse functions but are not seem to be essential for life because their gene’s deletion did not result in cellular death (Starai et al., 2002; Li et al., 2010, 2020; Liu et al., 2018a). However, we rarely know about deacetylases in diazotrophic organisms to date. We intend to investigate their roles in regulating nitrogen fixation and other metabolic pathways in the future.

## Conclusion

In summary, our work demonstrates the overall protein expression patterns and their PTM (acetylation)-based regulation under nitrogen fixation in *Z. mobilis*. Using a mass spectrometry-based proteomics approach, we performed a genome-wide analysis of proteome and protein acetylation in *Z. mobilis* and established its first acetylome under the N_2_-fixing and non-fixing conditions. The findings that we have presented here provide new knowledge of specific proteins and their associated cellular processes and pathways that may be regulated by protein acetylation in *Z. mobilis*. Functional validation of these acetylated proteins should be the focus of future research. The results of this study will serve as a baseline for further research in the area.

## Data Availability Statement

The datasets presented in this study can be found in online repositories. The names of the repository/repositories and accession number(s) can be found at: https://www.ebi.ac.uk/pride/, PXD027418.

## Author Contributions

BW and MH designed the study. YH, MC, and BW prepared samples for analysis. AN, XG, BW, and SK analyzed the data. AN and SK wrote the manuscript. BW and MH critically revised the manuscript. All authors contributed to the article and approved the submitted version.

## Funding

The study was supported by the National Natural Science Foundation of China (32070036), the Central Public-interest Scientific Institution Basal Research Fund (Y2019LM02), and the Local financial funds of National Agricultural Science and Technology Center (NASC2020AR02).

## Conflict of Interest

The authors declare that the research was conducted in the absence of any commercial or financial relationships that could be construed as a potential conflict of interest.

## Publisher’s Note

All claims expressed in this article are solely those of the authors and do not necessarily represent those of their affiliated organizations, or those of the publisher, the editors and the reviewers. Any product that may be evaluated in this article, or claim that may be made by its manufacturer, is not guaranteed or endorsed by the publisher.

## Footnotes

^1^ https://www.maxquant.org/

^2^ http://www.ebi.ac.uk/GOA/

^3^ https://swissmodel.expasy.org/

## Supplementary Material

Supplementary Figure S1 Hypothetical protein structure of NifH of *Zymomonas mobilis*. **(A)** Surface model. **(B)** Ribbon representation. **(C)** The acetylated residue K12 is shown in enlarged.

Supplementary Figure S2 Hypothetical protein structure of NifK of *Zymomonas mobilis*. **(A)** Surface model. **(B)** Ribbon representation. **(C)** The acetylated residue K395 is shown in enlarged.

Supplementary Figure S3 Ribbon representation of X-ray diffraction-based crystal structure of nitrogenase complex of *Azotobacter vinelandii* (PDB accession 4WZB). Corresponding K-residues for nitrogenase proteins of *Zymomonas mobilis* are highlighted.

## References

[ref1] Alencar V. C. Silva J. Vilas Boas R. O. Farnézio V. M. de Maria Y. Aciole Barbosa D.. (2021). The quorum sensing auto-inducer 2 (AI-2) stimulates nitrogen fixation and favors ethanol production over biomass accumulation in *Zymomonas mobilis*. Int. J. Mol. Sci. 22:5628. doi: 10.3390/ijms22115628, PMID: 34073173 PMC8198075

[ref2] Bai F. W. Anderson W. A. Moo-Young M. (2008). Ethanol fermentation technologies from sugar and starch feedstocks. Biotechnol. Adv. 26, 89–105. doi: 10.1016/j.biotechadv.2007.09.002, PMID: 17964107

[ref3] Bernal V. Castaño-Cerezo S. Gallego-Jara J. Écija-Conesa A. de Diego T. Iborra J. L.. (2014). Regulation of bacterial physiology by lysine acetylation of proteins. New Biotechnol. 31, 586–595. doi: 10.1016/j.nbt.2014.03.002, PMID: 24636882

[ref4] Bontemps-Gallo S. Gaviard C. Richards C. L. Kentache T. Raffel S. J. Lawrence K. A.. (2018). Global profiling of lysine acetylation in *Borrelia burgdorferi* B31 reveals its role in central metabolism. Front. Microbiol. 9:2036. doi: 10.3389/fmicb.2018.02036, PMID: 30233522 PMC6127242

[ref5] Bueno Batista M. Dixon R. (2019). Manipulating nitrogen regulation in diazotrophic bacteria for agronomic benefit. Biochem. Soc. Trans. 47, 603–614. doi: 10.1042/BST20180342, PMID: 30936245 PMC6490700

[ref6] Carabetta V. J. Cristea I. M. (2017). Regulation, function, and detection of protein acetylation in bacteria. J. Bacteriol. 199, e00107–e00117. doi: 10.1128/jb.00107-17, PMID: 28439035 PMC5527388

[ref7] Choudhary C. Kumar C. Gnad F. Nielsen M. L. Rehman M. Walther T. C.. (2009). Lysine acetylation targets protein complexes and co-regulates major cellular functions. Science 325, 834–840. doi: 10.1126/science.1175371, PMID: 19608861

[ref8] Christensen D. G. Baumgartner J. T. Xie X. Jew K. M. Basisty N. Schilling B.. (2019a). Mechanisms, detection, and relevance of protein acetylation in prokaryotes. MBio 10, e02708–e02718. doi: 10.1128/mBio.02708-18, PMID: 30967470 PMC6456759

[ref9] Christensen D. G. Xie X. Basisty N. Byrnes J. McSweeney S. Schilling B.. (2019b). Post-translational protein acetylation: An elegant mechanism for bacteria to dynamically regulate metabolic functions. Front. Microbiol. 10:1604. doi: 10.3389/fmicb.2019.01604, PMID: 31354686 PMC6640162

[ref10] Crosby H. A. Pelletier D. A. Hurst G. B. Escalante-Semerena J. C. (2012). System-wide studies of N-lysine acetylation in *Rhodopseudomonas palustris* reveal substrate specificity of protein acetyltransferases. J. Biol. Chem. 287, 15590–15601. doi: 10.1074/jbc.M112.352104, PMID: 22416131 PMC3346091

[ref11] Dixon R. (1998). The oxygen-responsive NIFL-NIFA complex: a novel two-component regulatory system controlling nitrogenase synthesis in gamma-proteobacteria. Arch. Microbiol. 169, 371–380. doi: 10.1007/s002030050585, PMID: 9560416

[ref12] Dixon R. Kahn D. (2004). Genetic regulation of biological nitrogen fixation. Nat. Rev. Microbiol. 2, 621–631. doi: 10.1038/nrmicro954, PMID: 15263897

[ref13] Doelle H. W. Kirk L. Crittenden R. Toh H. Doelle M. B. (1993). *Zymomonas Mobilis*—science and industrial application. Crit. Rev. Biotechnol. 13, 57–98. doi: 10.3109/07388559309069198, PMID: 8477453

[ref14] Drazic A. Myklebust L. M. Ree R. Arnesen T. (2016). The world of protein acetylation. Biochim. Biophys. Acta 1864, 1372–1401. doi: 10.1016/j.bbapap.2016.06.007, PMID: 27296530

[ref15] Ganusova E. E. Vo L. T. Abraham P. E. Yoder L. O. N. Hettich R. L. Alexandre G. (2021). The *Azospirillum brasilense* core chemotaxis proteins CheA1 and CheA4 link chemotaxis signaling with nitrogen metabolism. mSystems 6, e01354–e01320. doi: 10.1128/mSystems.01354-20, PMID: 33594007 PMC8561660

[ref16] Gardner J. G. Escalante-Semerena J. C. (2008). Biochemical and mutational analyses of AcuA, the acetyltransferase enzyme that controls the activity of the acetyl coenzyme a synthetase (AcsA) in *Bacillus subtilis*. J. Bacteriol. 190, 5132–5136. doi: 10.1128/JB.00340-08, PMID: 18487328 PMC2446994

[ref17] Glozak M. A. Sengupta N. Zhang X. Seto E. (2005). Acetylation and deacetylation of non-histone proteins. Gene 363, 15–23. doi: 10.1016/j.gene.2005.09.010, PMID: 16289629

[ref18] Hayden J. D. Brown L. R. Gunawardena H. P. Perkowski E. F. Chen X. Braunstein M. (2013). Reversible acetylation regulates acetate and propionate metabolism in *mycobacterium smegmatis*. Microbiology 159, 1986–1999. doi: 10.1099/mic.0.068585-0, PMID: 23813678 PMC3783017

[ref19] He M. X. Wu B. Qin H. Ruan Z. Y. Tan F. R. Wang J. L.. (2014). *Zymomonas mobilis*: a novel platform for future biorefineries. Biotechnol. Biofuels 7:101. doi: 10.1186/1754-6834-7-101, PMID: 25024744 PMC4094786

[ref20] Hebbes T. R. Thorne A. W. Crane-Robinson C. (1988). A direct link between core histone acetylation and transcriptionally active chromatin. EMBO J. 7, 1395–1402. doi: 10.1002/j.1460-2075.1988.tb02956.x, PMID: 3409869 PMC458389

[ref21] Hentchel K. L. Escalante-Semerena J. C. (2015). Acylation of biomolecules in prokaryotes: a widespread strategy for the control of biological function andmetabolic stress. Microbiol. Mol. Biol. Rev. 79, 321–346. doi: 10.1128/MMBR.00020-15, PMID: 26179745 PMC4503791

[ref22] Hoffmann M. C. Wagner E. Langklotz S. Pfänder Y. Hött S. Bandow J. E.. (2015). Proteome profiling of the *Rhodobacter capsulatus* molybdenum response reveals a role of IscN in nitrogen fixation by Fe-nitrogenase. J. Bacteriol. 198, 633–643. doi: 10.1128/JB.00750-15, PMID: 26644433 PMC4751811

[ref23] Hu L. I. Lima B. P. Wolfe A. J. (2010). Bacterial protein acetylation: the dawning of a new age. Mol. Microbiol. 77, 15–21. doi: 10.1111/j.1365-2958.2010.07204.x, PMID: 20487279 PMC2907427

[ref24] Huergo L. F. Pedrosa F. O. Muller-Santos M. Chubatsu L. S. Monteiro R. A. Merrick M.. (2012). PII signal transduction proteins: pivotal players in post-translational control of nitrogenase activity. Microbiology 158, 176–190. doi: 10.1099/mic.0.049783-0, PMID: 22210804

[ref25] Jiang P. Peliska J. A. Ninfa A. J. (1998). The regulation of *Escherichia coli* glutamine synthetase revisited: role of 2-Ketoglutarate in the regulation of glutamine synthetase adenylation state. Biochemistry 37, 12802–12810. doi: 10.1021/bi980666u, PMID: 9737857

[ref26] Kalnenieks U. (2006). “Physiology of *Zymomonas mobilis*: some unanswered questions,” in Advances in Microbial Physiology. ed. Poole R. K. (London: Academic Press), 73–117. 10.1016/S0065-2911(06)51002-1 17010696

[ref27] Kanehisa M. Goto S. (2000). KEGG: Kyoto encyclopedia of genes and genomes. Nucleic Acids Res. 28, 27–30. doi: 10.1093/nar/28.1.27, PMID: 10592173 PMC102409

[ref28] Kim S. C. Sprung R. Chen Y. Xu Y. Ball H. Pei J.. (2006). Substrate and functional diversity of lysine acetylation revealed by a proteomics survey. Mol. Cell 23, 607–618. doi: 10.1016/j.molcel.2006.06.026, PMID: 16916647

[ref29] Kremer T. A. LaSarre B. Posto A. L. McKinlay J. B. (2015). N2 gas is an effective fertilizer for bioethanol production by *Zymomonas mobilis*. Proc. Natl. Acad. Sci. U. S. A. 112, 2222–2226. doi: 10.1073/pnas.1420663112, PMID: 25646422 PMC4343144

[ref30] Lei S. Pulakat L. Gavini N. (1999). Genetic analysis of nif regulatory genes by utilizing the yeast two-hybrid system detected formation of a NifL-NifA complex that is implicated in regulated expression of nif genes. J. Bacteriol. 181, 6535–6539. doi: 10.1128/JB.181.20.6535-6539.1999, PMID: 10515947 PMC103792

[ref31] Li R. Gu J. Chen Y. Y. Xiao C. L. Wang L. W. Zhang Z. P.. (2010). CobB regulates *Escherichia coli* chemotaxis by deacetylating the response regulator CheY. Mol. Microbiol. 76, 1162–1174. doi: 10.1111/j.1365-2958.2010.07125.x, PMID: 20345663 PMC2883070

[ref32] Li P. Zhang H. Zhao G. P. Zhao W. (2020). Deacetylation enhances ParB-DNA interactions affecting chromosome segregation in *Streptomyces coelicolor*. Nucleic Acids Res. 48, 4902–4914. doi: 10.1093/nar/gkaa245, PMID: 32313947 PMC7229854

[ref33] Liimatta K. Flaherty E. Ro G. Nguyen D. K. Prado C. Purdy A. E. (2018). A putative acetylation system in *vibrio cholerae* modulates virulence in arthropod hosts. Appl. Environ. Microbiol. 84, e01113–e01118. doi: 10.1128/aem.01113-18, PMID: 30143508 PMC6193395

[ref34] Liu X. X. Shen M. J. Liu W. B. Ye B. C. (2018b). GlnR-mediated regulation of short-chain fatty acid assimilation in *mycobacterium smegmatis*. Front. Microbiol. 9:1311. doi: 10.3389/fmicb.2018.01311, PMID: 29988377 PMC6023979

[ref35] Liu W. Tan Y. Cao S. Zhao H. Fang H. Yang X.. (2018a). Protein acetylation mediated by YfiQ and CobB is involved in the virulence and stress response of *Yersinia pestis*. Infect. Immun. 86, e00224–e00218. doi: 10.1128/iai.00224-18, PMID: 29610260 PMC5964501

[ref36] Martien J. I. Hebert A. S. Stevenson D. M. Regner M. R. Khana D. B. Coon J. J.. (2019). Systems-level analysis of oxygen exposure in *Zymomonas mobilis*: implications for isoprenoid production. mSystems 4, e00284–e00218. doi: 10.1128/mSystems.00284-18, PMID: 30801024 PMC6372839

[ref37] Martinez-Argudo I. Little R. Dixon R. (2004a). A crucial arginine residue is required for a conformational switch in NifL to regulate nitrogen fixation in *Azotobacter vinelandii*. Proc. Natl. Acad. Sci. U. S. A. 101, 16316–16321. doi: 10.1073/pnas.0405312101, PMID: 15534211 PMC528952

[ref38] Martinez-Argudo I. Little R. Shearer N. Johnson P. Dixon R. (2004b). The NifL-NifA system: a multidomain transcriptional regulatory complex that integrates environmental signals. J. Bacteriol. 186, 601–610. doi: 10.1128/jb.186.3.601-610.2004, PMID: 14729684 PMC321506

[ref39] Marx H. Minogue C. E. Jayaraman D. Richards A. L. Kwiecien N. W. Siahpirani A. F.. (2016). A proteomic atlas of the legume *Medicago truncatula* and its nitrogen-fixing endosymbiont *Sinorhizobium meliloti*. Nat. Biotechnol. 34, 1198–1205. doi: 10.1038/nbt.3681, PMID: 27748755 PMC8557956

[ref40] Masepohl B. Hallenbeck P. C. (2010). Nitrogen and molybdenum control of nitrogen fixation in the phototrophic bacterium *Rhodobacter capsulatus*. Adv. Exp. Med. Biol. 675, 49–70. doi: 10.1007/978-1-4419-1528-3_4, PMID: 20532735

[ref41] Moure V. R. Costa F. F. Cruz L. M. Pedrosa F. O. Souza E. M. Li X. D.. (2015). Regulation of nitrogenase by reversible mono-ADP-ribosylation. Curr. Top. Microbiol. Immunol. 384, 89–106. doi: 10.1007/82_2014_380, PMID: 24934999

[ref42] Nakayasu E. S. Burnet M. C. Walukiewicz H. E. Wilkins C. S. Shukla A. K. Brooks S.. (2017). Ancient regulatory role of lysine acetylation in central metabolism. MBio 8, e01894–e01817. doi: 10.1128/mBio.01894-17, PMID: 29184018 PMC5705920

[ref43] Nordlund S. Högbom M. (2013). ADP-ribosylation, a mechanism regulating nitrogenase activity. FEBS J. 280, 3484–3490. doi: 10.1111/febs.12279, PMID: 23574616

[ref44] Ouidir T. Kentache T. Hardouin J. (2016). Protein lysine acetylation in bacteria: current state of the art. Proteomics 16, 301–309. doi: 10.1002/pmic.201500258, PMID: 26390373

[ref45] Palamae S. Choorit W. Chatsungnoen T. Chisti Y. (2020). Simultaneous nitrogen fixation and ethanol production by *Zymomonas mobilis*. J. Biotechnol. 314-315, 41–52. doi: 10.1016/j.jbiotec.2020.03.016, PMID: 32259548

[ref46] Panesar P. S. Marwaha S. S. Kennedy J. F. (2006). *Zymomonas mobilis*: an alternative ethanol producer. J. Chem. Technol. Biotechnol. 81, 623–635. doi: 10.1002/jctb.1448

[ref47] Perry S. Shearer N. Little R. Dixon R. (2005). Mutational analysis of the nucleotide-binding domain of the anti-activator NifL. J. Mol. Biol. 346, 935–949. doi: 10.1016/j.jmb.2004.12.033, PMID: 15701508

[ref48] Ree R. Varland S. Arnesen T. (2018). Spotlight on protein N-terminal acetylation. Exp. Mol. Med. 50, 1–13. doi: 10.1038/s12276-018-0116-z, PMID: 30054468 PMC6063853

[ref49] Ren J. Sang Y. Lu J. Yao Y. F. (2017). Protein acetylation and its role in bacterial virulence. Trends Microbiol. 25, 768–779. doi: 10.1016/j.tim.2017.04.001, PMID: 28462789

[ref50] Reverdy A. Chen Y. Hunter E. Gozzi K. Chai Y. (2018). Protein lysine acetylation plays a regulatory role in *Bacillus subtilis* multicellularity. PLoS One 13:e0204687. doi: 10.1371/journal.pone.0204687, PMID: 30265683 PMC6161898

[ref51] Sang Y. Ren J. Ni J. Tao J. Lu J. Yao Y. F. (2016). Protein acetylation is involved in *Salmonella enterica* serovar Typhimurium virulence. J. Infect. Dis. 213, 1836–1845. doi: 10.1093/infdis/jiw028, PMID: 26810370

[ref52] Sarkar A. Köhler J. Hurek T. Reinhold-Hurek B. (2012). A novel regulatory role of the Rnf complex of *Azoarcus* sp. strain BH72. Mol. Microbiol. 83, 408–422. doi: 10.1111/j.1365-2958.2011.07940.x, PMID: 22188282

[ref53] Schmitz R. A. Klopprogge K. Grabbe R. (2002). Regulation of nitrogen fixation in *Klebsiella pneumoniae* and *Azotobacter vinelandii*: NifL, transducing two environmental signals to the nif transcriptional activator NifA. J. Mol. Microbiol. Biotechnol. 4, 235–242. PMID: 11931553

[ref54] Sprenger G. A. (1996). Carbohydrate metabolism in *Zymomonas mobilis*: a catabolic highway with some scenic routes. FEMS Microbiol. Lett. 145, 301–307. doi: 10.1111/j.1574-6968.1996.tb08593.x

[ref55] Starai V. J. Celic I. Cole R. N. Boeke J. D. Escalante-Semerena J. C. (2002). Sir2-dependent activation of acetyl-CoA synthetase by deacetylation of active lysine. Science 298, 2390–2392. doi: 10.1126/science.1077650, PMID: 12493915

[ref56] Suyal D. C. Kumar S. Yadav A. Shouche Y. Goel R. (2017). Cold stress and nitrogen deficiency affected protein expression of psychrotrophic *dyadobacter psychrophilus* B2 and *Pseudomonas jessenii* MP1. Front. Microbiol. 8:430. doi: 10.3389/fmicb.2017.00430, PMID: 28352263 PMC5348510

[ref57] Szklarczyk D. Franceschini A. Wyder S. Forslund K. Heller D. Huerta-Cepas J.. (2015). STRING v10: protein-protein interaction networks, integrated over the tree of life. Nucleic Acids Res. 43, D447–D452. doi: 10.1093/nar/gku1003, PMID: 25352553 PMC4383874

[ref58] Tanabe M. Kanehisa M. (2012). Using the KEGG database resource. Curr. Protoc. Bioinformatics. 38, 1.12.1–1.12.43. doi: 10.1002/0471250953.bi0112s38, PMID: 22700311

[ref59] Tatli M. Hebert A. S. Coon J. J. Amador-Noguez D. (2019). Genome wide phosphoproteome analysis of *Zymomonas mobilis* under anaerobic, aerobic, and N_2_-fixing conditions. Front. Microbiol. 10:1986. doi: 10.3389/fmicb.2019.01986, PMID: 31551951 PMC6737584

[ref60] Todhanakasem T. Wu B. Simeon S. (2020). Perspectives and new directions for bioprocess optimization using *Zymomonas mobilis* in the ethanol production. World J. Microbiol. Biotechnol. 36:112. doi: 10.1007/s11274-020-02885-4, PMID: 32656581

[ref61] Tsoy O. V. Ravcheev D. A. Čuklina J. Gelfand M. S. (2016). Nitrogen fixation and molecular oxygen: comparative genomic reconstruction of transcription regulation in Alphaproteobacteria. Front. Microbiol. 7:1343. doi: 10.3389/fmicb.2016.01343, PMID: 27617010 PMC4999443

[ref62] Tucker A. C. Escalante-Semerena J. C. (2013). Acetoacetyl-CoA synthetase activity is controlled by a protein acetyltransferase with unique domain organization in *Streptomyces lividans*. Mol. Microbiol. 87, 152–167. doi: 10.1111/mmi.12088, PMID: 23199287 PMC3535548

[ref63] VanDrisse C. M. Escalante-Semerena J. C. (2019). Protein acetylation in bacteria. Annu. Rev. Microbiol. 73, 111–132. doi: 10.1146/annurev-micro-020518-115526, PMID: 31091420 PMC6736716

[ref64] Varley J. B. Wang Y. Chan K. Studt F. Nørskov J. K. (2015). Mechanistic insights into nitrogen fixation by nitrogenase enzymes. Phys. Chem. Chem. Phys. 17, 29541–29547. doi: 10.1039/C5CP04034E, PMID: 26366854

[ref65] Wang Y. Li P. Cao X. Wang X. Zhang A. Li X. (2009). Identification and expression analysis of miRNAs from nitrogen-fixing soybean nodules. Biochem. Biophys. Res. Commun. 378, 799–803. doi: 10.1016/j.bbrc.2008.11.140, PMID: 19084500

[ref66] Wang Q. Zhang Y. Yang C. Xiong H. Lin Y. Yao J.. (2010). Acetylation of metabolic enzymes coordinates carbon source utilization and metabolic flux. Science 327, 1004–1007. doi: 10.1126/science.1179687, PMID: 20167787 PMC4183141

[ref67] Wolfe A. J. (2016). Bacterial protein acetylation: new discoveries unanswered questions. Curr. Genet. 62, 335–341. doi: 10.1007/s00294-015-0552-4, PMID: 26660885 PMC4826803

[ref68] Xie Z. Ulrich L. E. Zhulin I. B. Alexandre G. (2010). PAS domain containing chemoreceptor couples dynamic changes in metabolism with chemotaxis. Proc. Natl. Acad. Sci. U. S. A. 107, 2235–2240. doi: 10.1073/pnas.0910055107, PMID: 20133866 PMC2836669

[ref69] Xiong Y. Guan K. L. (2012). Mechanistic insights into the regulation of metabolic enzymes by acetylation. J. Cell Biol. 198, 155–164. doi: 10.1083/jcb.201202056, PMID: 22826120 PMC3410420

[ref70] Yan Y. Ping S. Peng J. Han Y. Li L. Yang J.. (2010). Global transcriptional analysis of nitrogen fixation and ammonium repression in root-associated *Pseudomonas stutzeri* A1501. BMC Genomics 11:11. doi: 10.1186/1471-2164-11-11, PMID: 20053297 PMC2820453

[ref71] Yang S. Fei Q. Zhang Y. Contreras L. M. Utturkar S. M. Brown S. D.. (2016). *Zymomonas mobilis* as a model system for production of biofuels and biochemicals. Microb. Biotechnol. 9, 699–717. doi: 10.1111/1751-7915.12408, PMID: 27629544 PMC5072187

[ref72] Yang X. J. Seto E. (2008). Lysine acetylation: codified crosstalk with other posttranslational modifications. Mol. Cell 31, 449–461. doi: 10.1016/j.molcel.2008.07.002, PMID: 18722172 PMC2551738

[ref73] Yang S. Tschaplinski T. J. Engle N. L. Carroll S. L. Martin S. L. Davison B. H.. (2009). Transcriptomic and metabolomic profiling of *Zymomonas mobilis* during aerobic and anaerobic fermentations. BMC Genomics 10:34. doi: 10.1186/1471-2164-10-34, PMID: 19154596 PMC2651186

[ref74] Yang S. Vera J. M. Grass J. Savvakis G. Moskvin O. V. Yang Y.. (2018). Complete genome sequence and the expression pattern of plasmids of the model ethanologen *Zymomonas mobilis* ZM4 and its xylose-utilizing derivatives 8b and 2032. Biotechnol. Biofuels 11:125. doi: 10.1186/s13068-018-1116-x, PMID: 29743953 PMC5930841

[ref75] You D. Wang M. M. Ye B. C. (2017). Acetyl-CoA synthetases of *Saccharopolyspora erythraea* are regulated by the nitrogen response regulator GlnR at both transcriptional and post-translational levels. Mol. Microbiol. 103, 845–859. doi: 10.1111/mmi.13595, PMID: 27987242

[ref76] You D. Yin B. C. Li Z. H. Zhou Y. Yu W. B. Zuo P.. (2016). Sirtuin-dependent reversible lysine acetylation of glutamine synthetases reveals an autofeedback loop in nitrogen metabolism. Proc. Natl. Acad. Sci. U. S. A. 113, 6653–6658. doi: 10.1073/pnas.1525654113, PMID: 27247389 PMC4914186

[ref77] Yu C. S. Chen Y. C. Lu C. H. Hwang J. K. (2006). Prediction of protein subcellular localization. Proteins 64, 643–651. doi: 10.1002/prot.21018, PMID: 16752418

[ref78] Yu B. J. Kim J. A. Moon J. H. Ryu S. E. Pan J. G. (2008). The diversity of lysine-acetylated proteins in *Escherichia coli*. J. Microbiol. Biotechnol. 18, 1529–1536. PMID: 18852508

[ref79] Zhan Y. Deng Z. Yan Y. Zhang H. Lu C. Yang Z.. (2019). NfiR, a new regulatory noncoding RNA (ncRNA), is required in concert with the NfiS ncRNA for optimal expression of nitrogenase genes in *Pseudomonas stutzeri* A1501. Appl. Environ. Microbiol. 85, e00762–e00719. doi: 10.1128/aem.00762-19, PMID: 31076427 PMC6606865

[ref80] Zhan Y. Yan Y. Deng Z. Chen M. Lu W. Lu C.. (2016). The novel regulatory ncRNA, NfiS, optimizes nitrogen fixation via base pairing with the nitrogenase gene *nifK* mRNA in *pseudomonas stutzeri* A1501. Proc. Natl. Acad. Sci. U. S. A. 113, E4348–E4356. doi: 10.1073/pnas.1604514113, PMID: 27407147 PMC4968741

[ref81] Zhang Y. Pohlmann E. L. Roberts G. P. (2005). GlnD is essential for NifA activation, NtrB/NtrC-regulated gene expression, and posttranslational regulation of nitrogenase activity in the photosynthetic, nitrogen-fixing bacterium *Rhodospirillum rubrum*. J. Bacteriol. 187, 1254–1265. doi: 10.1128/JB.187.4.1254-1265.2005, PMID: 15687189 PMC545621

[ref82] Zhang T. Yan Y. He S. Ping S. Alam K. M. Han Y.. (2012). Involvement of the ammonium transporter AmtB in nitrogenase regulation and ammonium excretion in *Pseudomonas stutzeri* A1501. Res. Microbiol. 163, 332–339. doi: 10.1016/j.resmic.2012.05.002, PMID: 22659337

[ref83] Zhao S. Xu W. Jiang W. Yu W. Lin Y. Zhang T.. (2010). Regulation of cellular metabolism by protein lysine acetylation. Science 327, 1000–1004. doi: 10.1126/science.1179689, PMID: 20167786 PMC3232675

