# Supplementary figures and images for: Genome-Wide Analyses of Proteome and Acetylome in Zymomonas mobilis Under N2-Fixing Condition
Source: Front Microbiol. 2021 Oct 7;12:740555. doi: 10.3389/fmicb.2021.740555 (PMC8600466; doi:10.3389/fmicb.2021.740555)

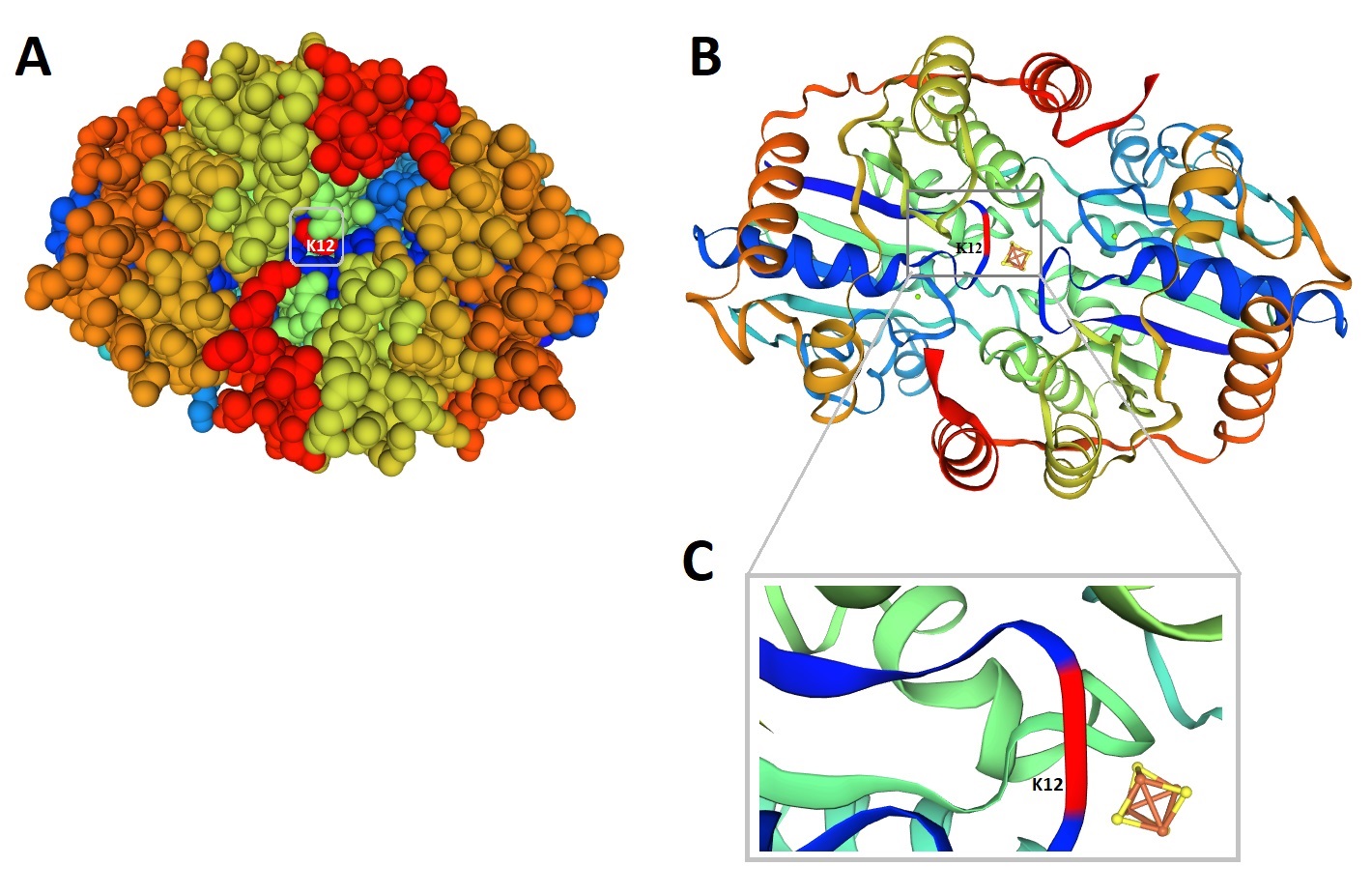

Supplement: Supplementary Figure S1 — Hypothetical protein structure of NifH of Zymomonas mobilis. (A) Surface model. (B) Ribbon representation. (C) The acetylated residue K12 is shown in enlarged. [file Image_1.JPEG]

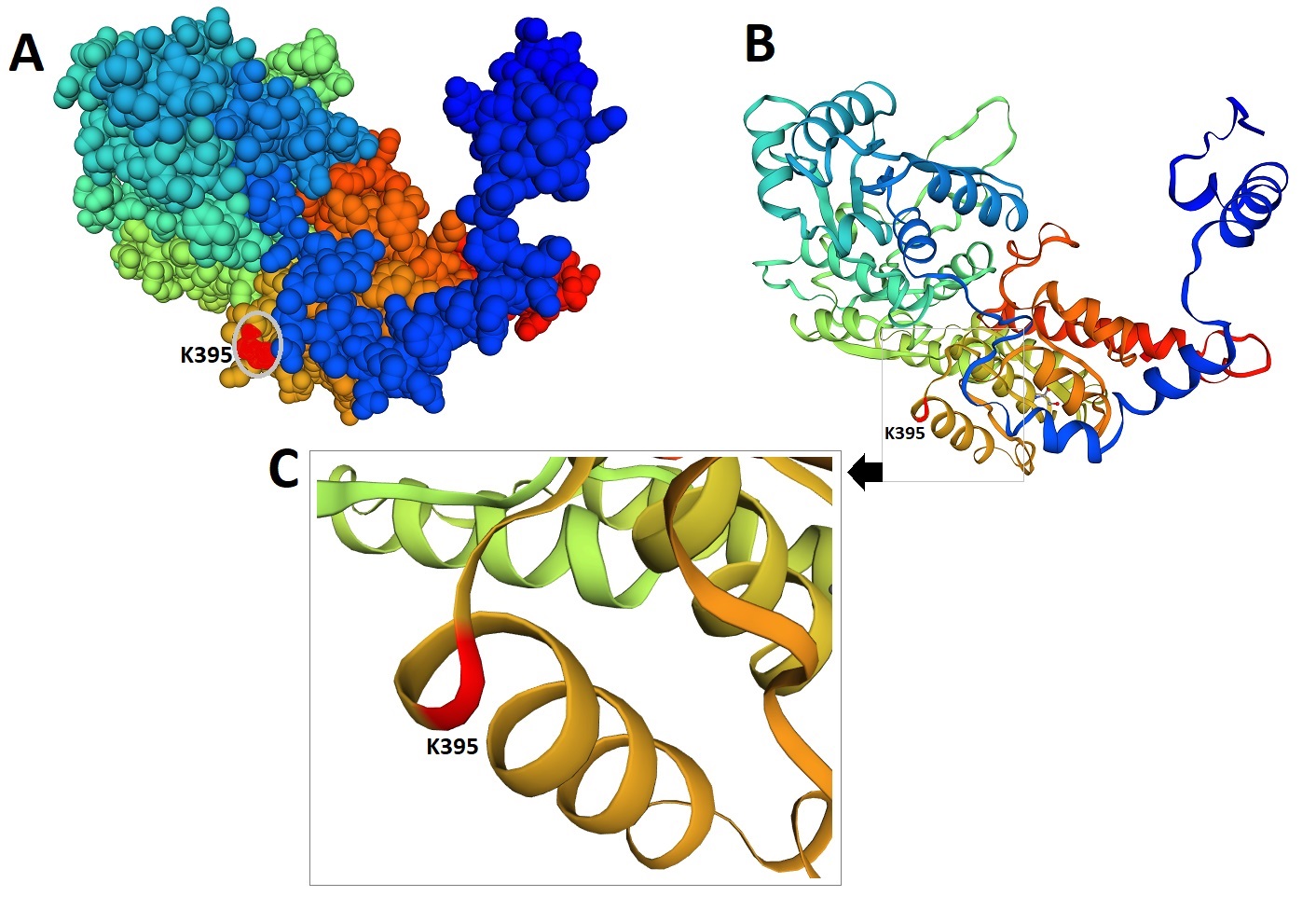

Supplement: Supplementary Figure S2 — Hypothetical protein structure of NifK of Zymomonas mobilis. (A) Surface model. (B) Ribbon representation. (C) The acetylated residue K395 is shown in enlarged. [file Image_2.JPEG]

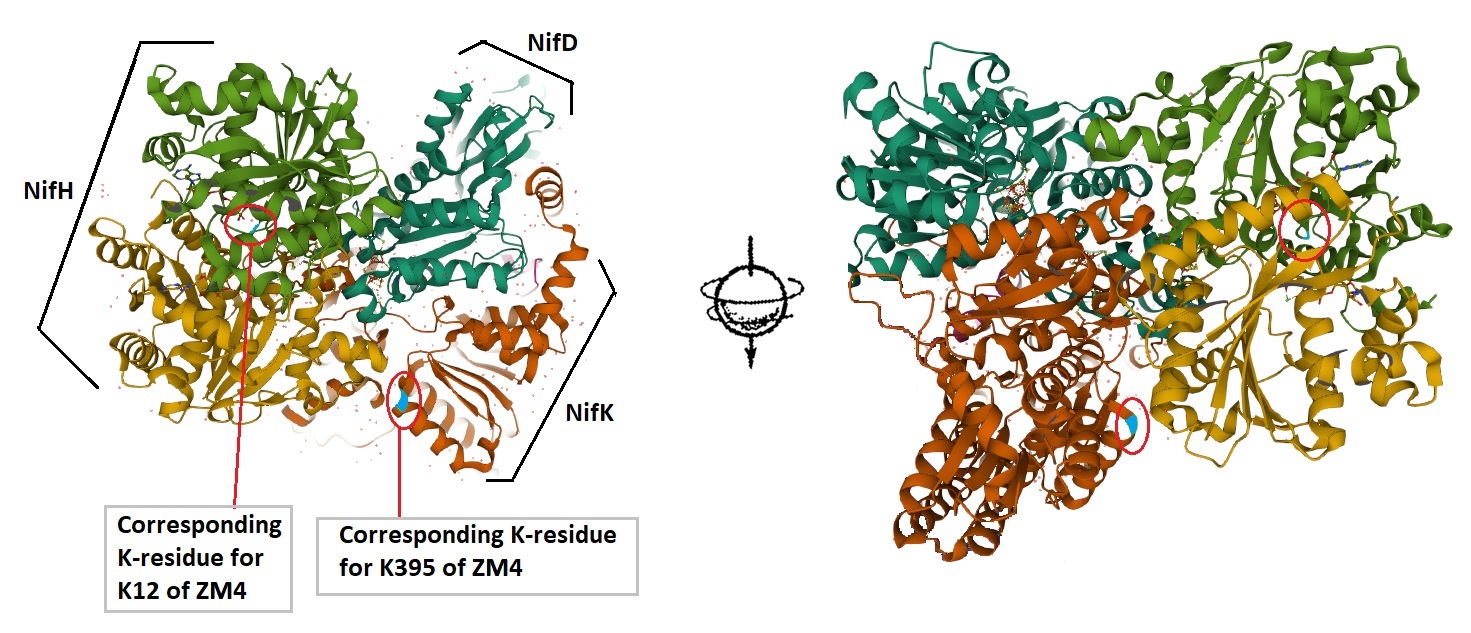

Supplement: Supplementary Figure S3 — Ribbon representation of X-ray diffraction-based crystal structure of nitrogenase complex of Azotobacter vinelandii (PDB accession 4WZB). Corresponding K-residues for nitrogenase proteins of Zymomonas mobilis are highlighted. [file Image_3.JPEG]
